# Sensory Reinforced Corticostriatal Plasticity

**DOI:** 10.2174/1570159X21666230801110359

**Published:** 2023-08-29

**Authors:** Nicolas Vautrelle, Véronique Coizet, Mariana Leriche, Lionel Dahan, Jan M. Schulz, Yan-Feng Zhang, Abdelhafid Zeghbib, Paul G. Overton, Enrico Bracci, Peter Redgrave, John N.J. Reynolds

**Affiliations:** 1 Department of Anatomy, Brain Health Research Centre, University of Otago, Dunedin 9054, New Zealand;; 2 Department of Psychology, University of Sheffield, Sheffield, S10 2TP, UK;; 3 Institut des Neurosciences de Grenoble, Université Joseph Fourier, Inserm, U1216, 38706 La Tronche Cedex, France;; 4 Centre de Recherches sur la Cognition Animale, Université de Toulouse, UPS, 118 Route de Narbonne, F-31062 Toulouse Cedex 9, France;; 5 Department of Biomedicine, University of Basel, CH - 4056 Basel, Switzerland;; 6 Department of Clinical and Biomedical Sciences, University of Exeter Medical School, Hatherly Laboratories, Exeter EX4 4PS, United Kingdom

**Keywords:** Corticostriatal, plasticity, timing, dopamine, sensory, reinforcement

## Abstract

**Background:**

Regional changes in corticostriatal transmission induced by phasic dopaminergic signals are an essential feature of the neural network responsible for instrumental reinforcement during discovery of an action. However, the timing of signals that are thought to contribute to the induction of corticostriatal plasticity is difficult to reconcile within the framework of behavioural reinforcement learning, because the reinforcer is normally delayed relative to the selection and execution of causally-related actions.

**Objective:**

While recent studies have started to address the relevance of delayed reinforcement signals and their impact on corticostriatal processing, our objective was to establish a model in which a sensory reinforcer triggers appropriately delayed reinforcement signals relayed to the striatum *via* intact neuronal pathways and to investigate the effects on corticostriatal plasticity.

**Methods:**

We measured corticostriatal plasticity with electrophysiological recordings using a light flash as a natural sensory reinforcer, and pharmacological manipulations were applied in an *in vivo* anesthetized rat model preparation.

**Results:**

We demonstrate that the spiking of striatal neurons evoked by single-pulse stimulation of the motor cortex can be potentiated by a natural sensory reinforcer, operating through intact afferent pathways, with signal timing approximating that required for behavioural reinforcement. The pharmacological blockade of dopamine receptors attenuated the observed potentiation of corticostriatal neurotransmission.

**Conclusion:**

This novel *in vivo* model of corticostriatal plasticity offers a behaviourally relevant framework to address the physiological, anatomical, cellular, and molecular bases of instrumental reinforcement learning.

## 1 INTRODUCTION

A century ago, Thorndike’s cat was confined in a cage until, unwittingly, it pressed against a pedal which opened the cage door, giving the animal access to a piece of fish [1]. With repeated trials, the animal gradually learned what it had to do, so when placed in the cage again, it was able to select the newly acquired action of pedal pressing and gain immediate access to the fish. This first formal demonstration of instrumental conditioning exemplifies reinforcement-driven action acquisition where an unexpected sensory reinforcer (the cage-door opening) enables relevant neural systems to converge onto the causal aspects of the cat’s behaviour, the pedal press. Accumulating empirical evidence points to the basal ganglia, specifically the dorsal striatum, playing a critical role in such reinforcement-driven action acquisition [2-6]. In most models of this process [7-10], signals assumed to represent behavioural options originating from the cerebral cortex induce patterns of activity in the striatum, which are differentially reinforced by consequent sensory events that evoke phasic signals from midbrain dopaminergic neurons. Phasic dopamine (DA) activity is evoked by unexpected, non-habituated sensory events [11-14], including those associated with reward [14-17]. Historically, two main experimental protocols have been used to investigate the biological mechanisms of corticostriatal plasticity: (i) high-frequency stimulation of afferent corticostriatal fibres in association with postsynaptic neuron firing [18]; and (ii) spike-timing-dependent-plasticity (STDP) protocols in which pre- and post-synaptic activity in striatal neurons is manipulated to demonstrate long-term changes in corticostriatal transmission [19-22]. These paradigms have shown that the timing of activation of the pre-and post-synaptic elements and the presence/absence of DA is critical for certain forms of corticostriatal plasticity [17, 18, 21, 22]. It has, however, been difficult to reconcile the timing aspects of early experimental protocols with behavioural reinforcement in which delayed reinforcing sensory signals (the cage door opening in the case of Thorndike’s cat) typically occur hundreds of milliseconds, sometimes seconds, after the relevant causal behaviour (the cat pushing the pedal) [23-26]. Many previous studies have investigated the impact of phasic dopaminergic signals on action selection during the execution of well-learned tasks [27-32]. However, studies investigating the relative timing of afferent cortical and dopaminergic signals on corticostriatal plasticity that may underlie action discovery are limited. For example, the timing of dopaminergic signals seems to be crucial for (i) modulating the structural plasticity of dendritic spines of medium spiny neurons (MSN) [33], (ii) STDP of corticostriatal synapses on D1 and D2-type receptor-expressing MSN [34-36], and (iii) the interaction with cholinergic signalling in the induction of short-term corticostriatal potentiation [37]. However, a model of lasting corticostriatal plasticity in which the temporal dynamic of signals likely to converge within the striatum can be systematically manipulated at timescales consistent with action discovery remains to be interrogated. To address this issue, our strategy was to develop an *in vivo* preparation that permitted precise control over afferent signalling within the relevant neural network.

**Fig. (1):**
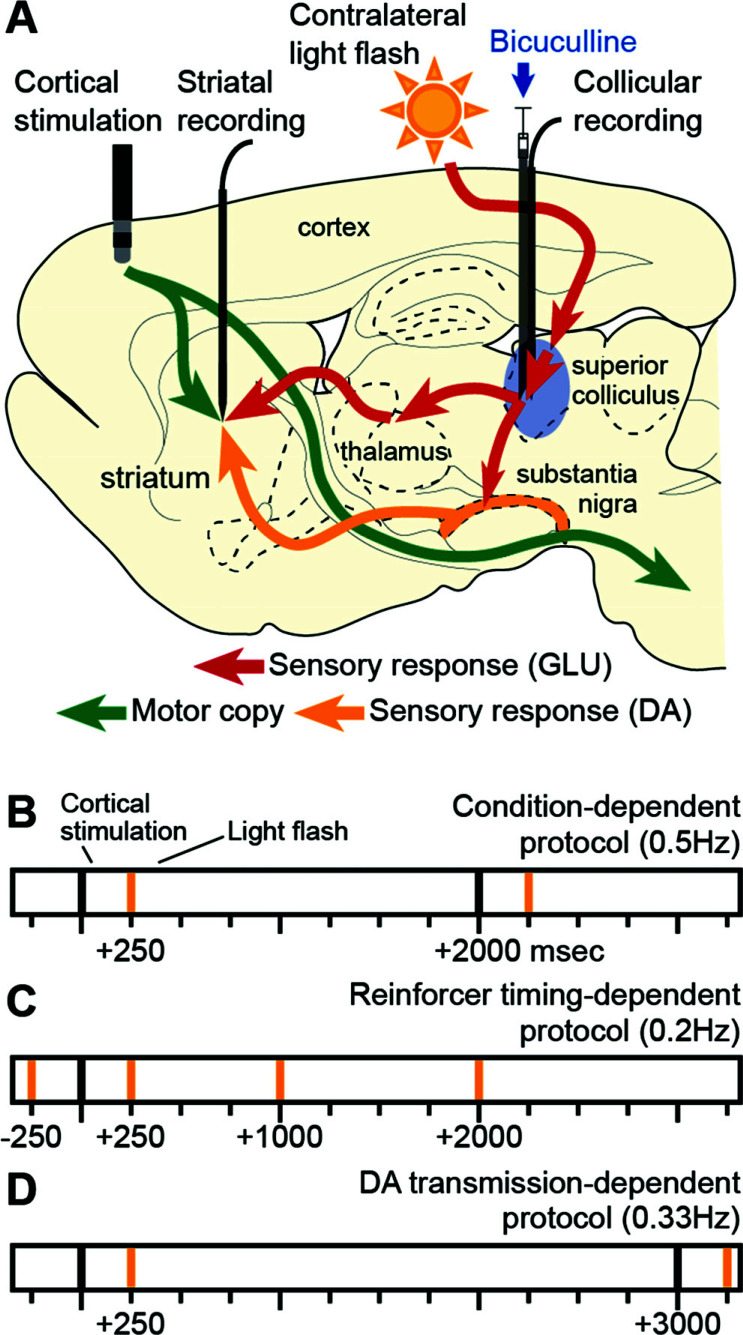
Experimental paradigm and protocols. (**A**) An experimental paradigm to demonstrate sensory-reinforced corticostriatal plasticity. (i) Single electrical pulses were delivered to ipsilateral motor cortex (0.1 ms; 0.2-1.0 mA; 0.2-0.5 Hz). Sensory reinforcement relayed *via* the thalamostriatal (ii) and nigrostriatal (iii) projections was provided by a contralateral light flash delayed by 250 ms in the presence of a disinhibitory injection of the GABA_A_ antagonist (bicuculline, 50 ng/ 500 nl) into the superior colliculus. (**B**, **C & D**) The timing of stimuli in the three experimental protocols. (**B**) In the first set of experiments, motor cortex stimulation (black bars) was delivered with an average frequency of 0.5 Hz (ISI 30% jittered - range 1.4 to 2.6 sec). A reinforcing whole-field light flash (yellow bars) delayed by + 250 ms was paired with each cortical stimulation. (**C**) In the second set of experiments, cortical stimulation (black bar) was applied at 0.2 Hz on average (ISI 30% jittered). The timing of the reinforcing light flash (yellow bars) was systematically varied to occur either before (-250 ms) or after (+250, +1000, +2000 ms) each cortical stimulation. (**D**) In a third set of experiments, cortical stimulation (black bars) was delivered at an average frequency of 0.33 Hz (ISI 30% jittered). A reinforcing whole-field light flash (yellow bars) delayed by +250 ms was paired with each cortical stimulation.

Based on our analysis of basal ganglia functional anatomy [5, 6] and following the neoHebbian three-factor learning rules [24, 25, 38], we sought to model three principal sources of input likely to be engaged during instrumental conditioning by a visual reinforcer (Fig. **1A**): (i) the afferent collateral fibres, branching from cortical motor projections to the brainstem, which ensure that the striatum receives a running copy of the motor commands directing behavioural output [39, 40]; (ii) the short-latency information signalling the occurrence of an unexpected, salient visual event, relayed *via* ascending glutamatergic thalamostriatal projections [40-43]; and (iii) the short-latency, visually-evoked, phasic DA input from substantia nigra [44], widely considered to act as a critical reinforcement signal for corticostriatal plasticity [15, 17]. Following the onset of a potentially reinforcing salient visual event, an important source of short-latency input to both nigral DA neurons and thalamic regions that project to the striatum is from branching tecto-nigral/tecto-thalamic fibres that originate from deep-layer neurons of the midbrain superior colliculus (Fig. **1A**) [45-47]. Earlier studies by our group have demonstrated that these bifurcating projections will ensure that a single reinforcing visual event can evoke near-simultaneous and potentially converging phasic inputs of DA and glutamate (GLU) into the striatum [43, 44]. Coincident DA and GLU input to the striatum has been shown to be essential for activating the plasticity marker ERK and expressing drug-induced locomotor sensitization [48] (Fig. **1**). With this point in mind, we exploited our knowledge of how to use a neutral stimulus (a light flash) repetitively to produce combined short-latency release of DA [44] and GLU [43] into the striatum *via* intact pathways in anaesthetized rats (Fig. **1A**). These procedures rely on the important discovery of Katsuta and Isa [49] who showed that a local injection of the GABAA antagonist bicuculline into the superior colliculus could restore visual responsiveness to deep layer neurons, previously rendered insensitive by anaesthesia. Therefore, the present study was designed to i) test whether sensory-reinforced corticostriatal plasticity could be demonstrated by pairing electrical stimulation of the motor cortex with simultaneous and appropriately timed sensory-evoked inputs from the thalamus (GLU) [43] and substantia nigra (DA) [44]; ii) test whether the temporal dynamics of the observed sensory-reinforced plasticity conformed to the timing of behavioural reinforcement learning; and iii) determine the extent to which intact dopaminergic neurotransmission is essential for this form of corticostriatal plasticity. In our model, the precisely controlled electrically-evoked input from the motor cortex takes the place of a motor command (*e.g*., a pedal press), which could be causally related to a consequent light flash (in the case of Thorndike’s cat, the door opening). Our prediction was that appropriate timing of the cortical-motor, and visually-evoked sensory inputs should induce prolonged reinforcement of the corticostriatal response in this potentially causal association [5, 10]. The demonstration of a novel, behaviourally relevant, *in vivo* model of sensory-reinforced corticostriatal plasticity, confirmed this prediction. Subsequent experiments showed that a pharmacological blockade of dopamine receptors partially suppressed the observed potentiation of corticostriatal transmission.

## 2 MATERIALS AND METHODS

### 2.1 Care of Animals

All animal husbandry and experimental procedures were performed in the UK with Govt. Home Office approval under section 5(4) of the Animals (Scientific Procedures) Acts 1986. In New Zealand, experiments were conducted in compliance with the Animal Welfare Act 1999. Experimental protocols also received prior approval from the relevant Institutional Ethics Committees.

## 2.2 Surgical Techniques

Seventy-six Hooded Lister and 9 Long Evans male rats (250-450 g) were prepared for electrophysiological recording under urethane anesthesia (1.25-2.0 g/kg). A concentric bipolar stimulating electrode (NEX-100, Rhodes Medical Instruments, Inc.) was introduced in the primary motor cortex (AP +3.7 to +2.2 mm, bregma; ML +2.0 to +3.0 mm, midline; DV -1.3 to -2.0 mm, dura). A tungsten microelectrode (A-M Systems, Inc., 2 MΩ) glued to a 30-gauge metallic injector needle filled with bicuculline methiodide (Sigma Aldrich, 100 ng/µl 0.9% saline) was placed vertically into the intermediate layers of the ipsilateral lateral superior colliculus (AP -6.3 to -7.3 mm, bregma; ML + 1.5 to 2.5 mm, midline; DV -4.5 to -5.3 mm, dura). An ipsilateral approach (angled 15° in the mediolateral plane; AP +0.2 to -0.8 mm, bregma; ML +2.0 to +3.5 mm, midline; DV -5.0 to -6.0 mm, dura) was used to position a multi-unit (2 MΩ tungsten or NeuroNexus, 16 channels) or single-unit microelectrode (6-13 MΩ glass pipette, internal solution: 0.5 M potassium acetate) into the striatal receptive field responsive to stimulation of the motor cortex ([40, 50] and Fig. **S1**). In the experiments where striatal microinjections of lidocaine (20-40 nl, 40 µg/µl, Sigma Aldrich) were made, a 30 µm diameter glass injection pipette was glued to the striatal single channel tungsten microelectrode.

## 2.3 Recording Techniques

A Micro 1401 hardware acquisition system connected to a standard PC running Spike 2 software (Cambridge Electronic Design) was used to sample striatal and collicular local field potential (filter setting: DC-50 Hz) and multi- or single-unit activity (filter setting: 0.2-15 kHz, sampling rate: 15 kHz). A System 3 modular rack-mount workstation (Tucker-Davis Technology) connected *via* a F15 Gigabit interface to a standard PC running a custom Matlab^TM^ script was used to sample striatal 16 channels multiunit activity (unfiltered signal, sampling rate: 25 kHz).

**Fig. (2):**
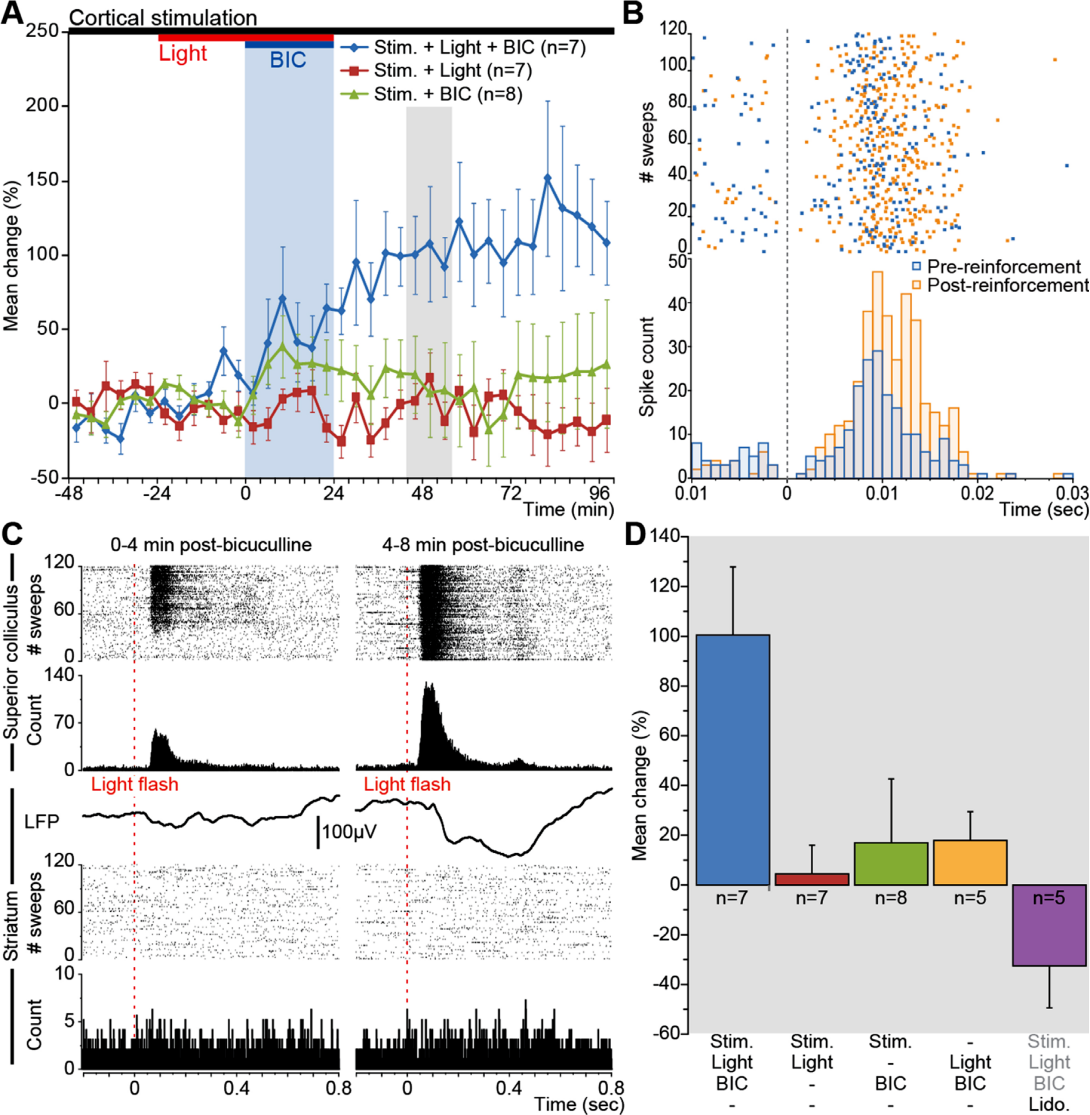
Sensory-reinforced corticostriatal plasticity. (**A**) Single cortical pulses (0.5 Hz) were presented throughout (black bar). Presentation of the reinforcing light flash (+250 ms) is indicated by the red bar. Dishinibition of the superior colliculus is indicated by the blue shading. Each point represents the mean change (%) in the magnitude of striatal multi-unit responses. (**B**) A single case example of striatal multi-unit potentiation (raster plots and associated peri-stimulus histograms) (**C**) Visual reinforcement failed to evoke spiking responses in the striatum. Bicuculline-induced restoration of visual responses to deep layer collicular neurons (top graphs); visually-evoked striatal local field potential (middle graphs); striatal multi-unit spiking (bottom graphs). (**D**) For all experimental conditions, mean change (%) in the magnitude of the cortical stimulus-evoked striatal responses 44-56 min after collicular disinhibition (grey shaded area in **A**). Experimental conditions are below figure.

In the first series of experiments (Figs. **1B** and **2**), multi-unit responses to ipsilateral motor cortex stimulation (single 100 µs duration pulse, 0.2-1.0 mA intensity, single pulse recurrence 0.5 Hz, 30% jittered) were recorded in the striatum and the superior colliculus. After recording 6 blocks of cortical stimulation-evoked responses (120 stimulations/block), each motor cortex stimulation was paired with a whole-field light flash (10 ms duration) delayed by +250 ms. The flash was delivered from a green LED (570 nm, 60 LUX) positioned 5 mm from the eye contralateral to the stimulation and recording electrodes. After recording 6 more stimulation blocks (120 stimulations/block), bicuculline methiodide was injected into the lateral part of the deep layers of the superior colliculus (0.5 µl, 1 µl/min). Disinhibition of the superior colliculus, assessed by online observation of a clear multi-unit response evoked by the light flash, typically lasted 10-20 min. When the disinhibitory effect of bicuculline had worn off, the light flash was discontinued. Recording of striatal and collicular responses to motor cortex stimulation continued for up to 3 h.

**Fig. (3):**
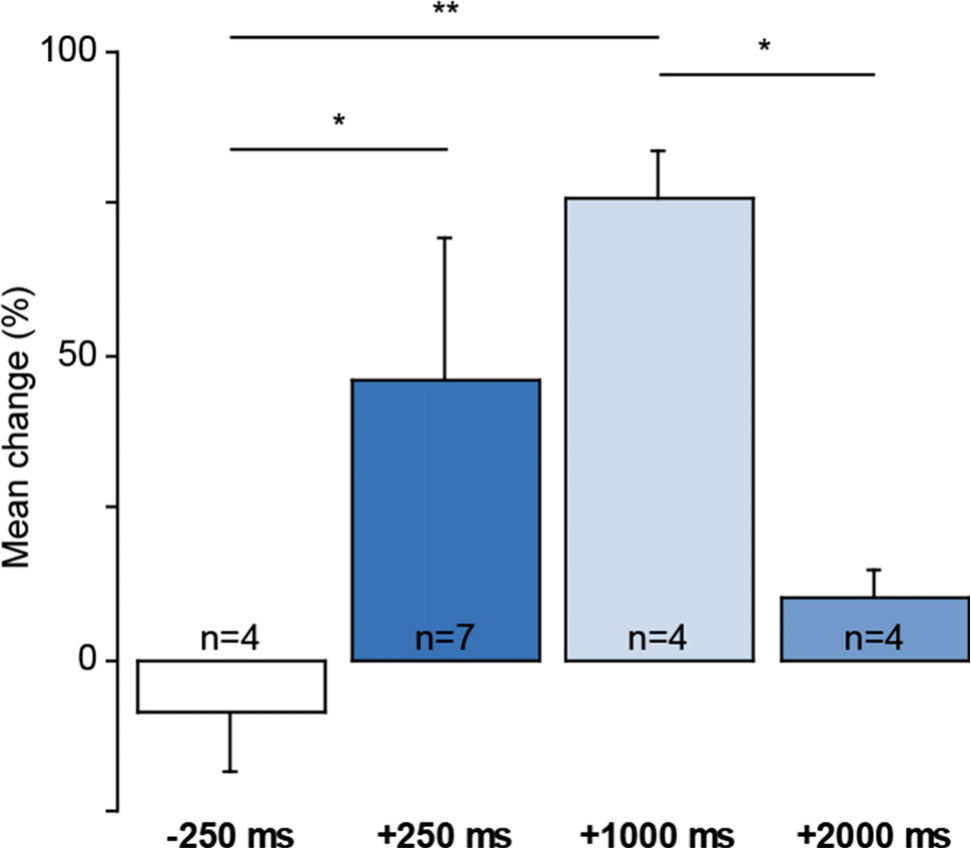
Sensory reinforcement within a behaviourally relevant time window. Only when light reinforcement was delivered +250 and +1000 ms after the cortical stimulus was significant potentiation of the evoked striatal response observed (Two-way ANOVA: F_3,15_ = 3.6; *p* < 0.04, Fisher’s PLSD test: *p < 0.05, **p < 0.01).

In the second set of experiments (Figs. **1C** and **3**), the ipsilateral single pulse cortical stimulation was delivered with a 0.2 Hz recurrence to accommodate our longer reinforcement delay of +2 sec. After recording 3 blocks of cortical stimulation-evoked responses (120 stimulations/block), each motor cortex stimulation was paired with a light flash presented either before (-250 ms, N = 7) or after (+250 ms, N = 4; +1000 ms, N = 4; or +2000 ms, N = 4) the cortical stimulation pulse. After recording 3 more stimulation blocks, bicuculline methiodide was injected into the lateral part of the deep layers of the superior colliculus (0.5 µl, 1 µl/min). Following the disinhibitory effect of bicuculline, the light was switched off, and striatal and collicular responses to motor cortex stimulation were recorded for up to 3 h.

In the third series of experiments (Figs. **1D** and **6**), multi-unit responses to ipsilateral motor cortex stimulation (0.33 Hz recurrence) were recorded in the striatum over 16 channels (Fig. **S1B**). After recording 4 blocks of cortical stimulation-evoked responses (120 stimulations/block), the animals received an i.p. injection of either saline (0.9%), D1-type dopamine receptor antagonist SCH 23390 hydrochloride (0.2 mg/kg, Sigma), D2-type dopamine receptor antagonist Sulpiride (30 mg/kg, Sigma) or both D1 and D2-type dopamine receptor antagonists. After recording 4 more stimulation blocks (24 mins), each motor cortex stimulation was paired with a light flash presented 250 ms after the cortical stimulation pulse. After recording 4 more blocks, bicuculline methiodide was injected into the lateral part of the deep layers of the superior colliculus (0.5 µl, 1 µl/min). Following the disinhibitory effect of bicuculline, the light was switched off, and striatal and collicular responses to motor cortex stimulation were recorded for up to 3 h.

**Fig. (4):**
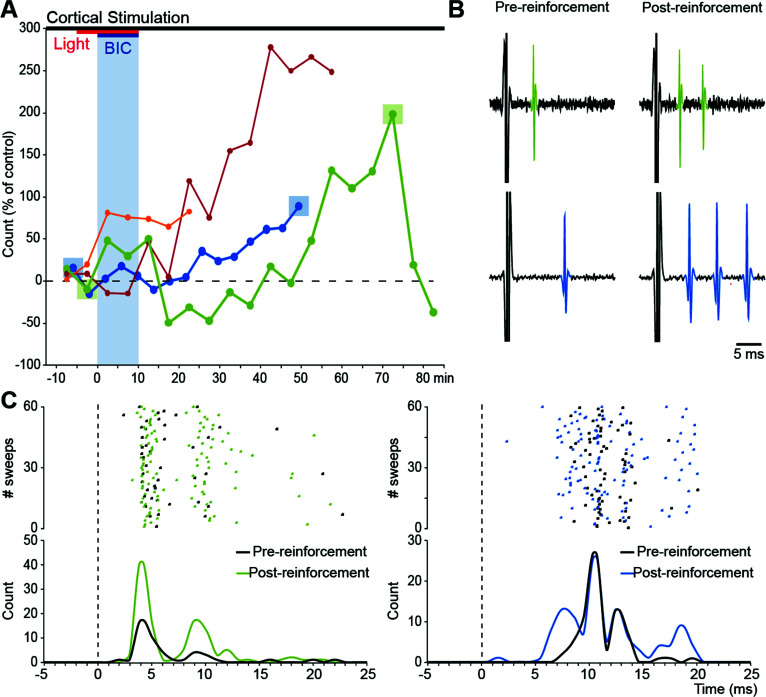
Changes in neuronal activity underlying sensory-reinforced corticostriatal plasticity. (**A**) Four examples of varied responses of individual neurons (different colored lines). For two neurons (dark blue and green lines), the squares mark the trials of cortical stimulation from which raster and histogram figures were calculated in C. (**B**) Examples of pre- and post-reinforcement activity of the two single units whose data are plotted in C. (**C**) In one case (green) spiking occurred more frequently at the same latencies after sensory reinforcement, while in the other case (blue) responses at some latencies remained unaltered while spiking at new shorter latencies appeared following potentiation.

During single-unit recording experiments (Fig. **4**), single pulse cortical stimulation of the motor cortex was delivered with a 0.2 Hz recurrence (0.5-1 mA, 0.1 to 0.25 ms) and paired with a light flash delayed by +250 ms. After recording one block of cortical stimulation-evoked responses (60 stimulations/block) and one block of cortical stimuli paired with the light flash, bicuculline methiodide (0.2-0.3 µl, 0.4 µl/ min) was injected in the lateral superior colliculus. Visual stimulation continued until the collicular disinhibition was no longer present. Recording of the response of the striatal neuron to motor cortex stimulation was maintained until the cell was lost (30-90 min). In some single-unit experiments, the stimulating electrode was placed in the contralateral motor cortex (AP 2.0 mm bregma; ML -1.6 mm midline; DV-2.3 mm, dura). The pattern of response plasticity was similar to that obtained using ipsilateral electrode placements; hence these experiments were considered together.

### 2.4 Histology

Following the experiment, animals were perfused intracardially with saline (0.9%), followed by paraformaldehyde (4%), and their brains were taken for histological analysis. Using standard immunohistochemical procedures, sections of cortical, striatal, and collicular tissues were reacted to reveal Fos-like immunoreactivity (rabbit polyclonal antibody, 1:20,000 dilution) evoked by electrical, sensory, and chemical stimulation. Fos-like immunoreactivity was only detected in the superior colliculus of animals that had received a bicuculline injection. The distribution of Fos-positive neurons was subjectively analysed to determine the extent of the collicular area activated by each bicuculline injection (Fig. **S2C**). Other sections were stained with cresyl-violet to verify the locations of the recording and stimulation sites (Figs. **S2**, **S3**, and **S4**).

### 2.5 Data Analysis

**Fig. (5):**
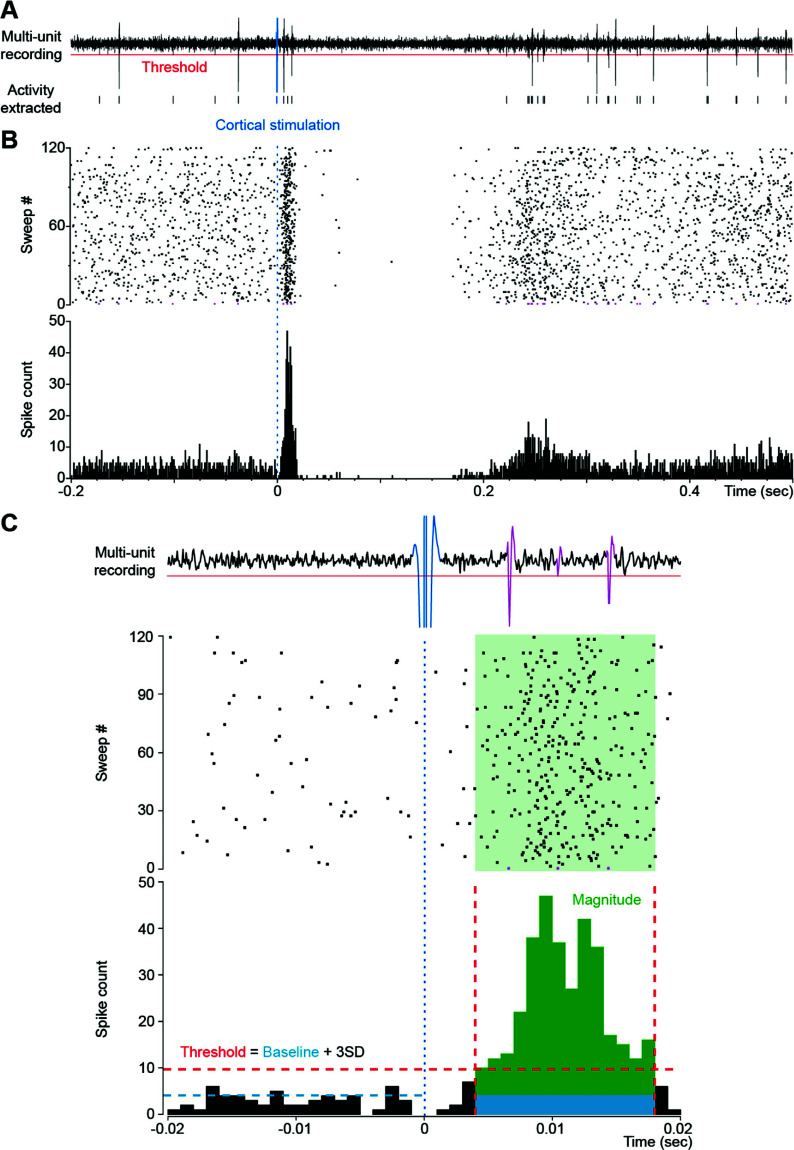
Analysis of multi-unit recording. (**A**) Multi-unit spike activity evoked by the cortical stimulus and the light flash were recorded locally in the striatum. (**B**) Data were processed in the form of spike-count rasters and peri-stimulus histograms. (**C**) Multi-unit striatal responses were recorded in successive blocks of 120 cortical stimulations. For each block multi-unit response characteristics were determined from the peri-stimulus histograms (bin width 1 ms). Response duration was determined by considering the consecutive bins when the firing rate exceeded 3SD (red dotted lines) over the mean base-line firing rate (blue dotted line). Response magnitude (green) for each block of 120 stimulations was recorded as the number of counts during the response minus the mean baseline count for the same period (blue).

Data were processed offline using CED Spike 2, Matlab^TM^ software, and custom scripts. Multi-unit activity was extracted from high-pass filtered waveforms by applying a threshold determined over the baseline recordings for each experiment to include a wide range of striatal neurons responsive to motor cortex stimulation (Fig. **5**). For both multi- and single-unit recordings, spike-count rasters and peri-stimulus time histograms were aligned on cortical stimulation onset (Figs. **5B** and **S7A**). For the first 2 series of experiments, a threshold value (mean frequency + three times the standard deviation of the mean frequency) was calculated over 500 ms of baseline spontaneous activity preceding the cortical stimulation (Fig. **5C**). The peak of the cortically-evoked response was then detected during the 50 ms following stimulation. The evoked-response onset and offset were defined as the time of the first bin to exceed or fall below the threshold before and after the peak, respectively. Response magnitude was defined as the number of spike counts during the evoked response minus the mean baseline count for the same period (Fig. **5C** green). The value for each block (120 cortical stimulations) was normalized for each subject as a percentage change relative to the mean value of the blocks obtained prior to the injection of bicuculline. For the third set of experiments, striatal responses to cortical stimulation were obtained by subtracting from each peri-stimulus time histogram its own mean spontaneous firing calculated over the 500 ms of spontaneous activity preceding the cortical stimulation (Fig. **S7A** – green line). An average baseline response to cortical stimulation was then calculated for each channel over the 8 blocks preceding the bicuculline injection (4 post-drug blocks of stimulation + 4 post-drug blocks of stimulation paired with light flash; Fig. **S7C** blue period and Fig. **S7B** blue traces). Over all channels, peaks of potentiation were then detected for each block. A peak of potentiation was detected (Fig. **S7B** red dots) if a bin value in the block response (Fig. **S7B** green trace) was greater than the sum of the same bin value in the average baseline response (Fig. **S7B** blue trace) plus two standard deviations (Fig. **S7B** blue shading). Potentiation peaks were then plotted against time over the experiment (Fig. **S7C**). A channel was considered potentiated if it met one of the following requirements: i. following bicuculline injection, potentiation peaks with similar latencies were detected over a minimum of 5 consecutive blocks, and such peaks were absent during the pre-drug period; or ii. following bicuculline injection, potentiation peaks with similar latencies were detected over seven or more consecutive blocks, and such peaks were absent during the pre-drug period.

The latency of the potentiated response was defined as the time between the electrical stimulation and the first bin of the potentiated response. The duration of the potentiated response was defined as the number of bins over which a potentiated response was observed. To determine the magnitude of potentiation, the spike-count values for each peak of potentiation were calculated as the difference between the bin value of the block response (green trace) and the bin value of the average baseline response (blue trace). The total magnitude of the potentiation of the response was then calculated by summing the spike counts of all peaks of potentiation. The average magnitude of the potentiation was calculated by dividing that sum by the duration of the potentiated response.

### 2.6 Statistical Analysis

Group comparison of cortical stimulation-induced striatal responses elicited over the full-time period were made using repeated measures ANOVA to separate group and time effects. A Mann-Whitney U test was used to compare over all experimental conditions and the non-normally distributed mean change (%) in striatal response magnitude data at 44-56 min after collicular disinhibition. Changes from baseline were assessed using a Wilcoxon matched-pairs-signed-rank and Kruskal-Wallis tests. Within-group effects were analysed using paired t-tests.

To assess the effect of the dopamine antagonist(s) on striatal responses to cortical stimulation (Pre-drug baseline period (purple) *vs*. Post-drug baseline period (blue) in Fig. (**S7C**), an ANOVA-like table with tests of random-effect terms (RANOVA) was used [51]. This statistic is employed as a measure of the size of the difference between the conditions. A Chi-Square test was used to determine the effect of the drug treatments on the proportion of electrode channels on which pairing induced significant potentiation. Significance was considered for two-tailed *p* values < 0.05.

## 3 RESULTS

To simulate motor-copy input to the striatum in a controlled manner, single electrical pulses (0.1 ms; 0.2-1.0 mA; 0.5 Hz) were delivered to the ipsilateral motor cortex (Figs. **S2A**, **S3A**, and **S4A**) and recordings made from neurons in the dorsal striatum (Figs. **S2B**, **S3B**, and **S4B**). A contralateral whole-field light flash provided sensory reinforcement in the presence of a disinhibitory injection of bicuculline (50 ng/500 nl) into the deep layers of the superior colliculus [49] (Figs. **S2C**, **S3C**, and **S4C**). We have shown this treatment ensures that each light flash can effectively activate nigral and thalamic input to the striatum over an extended period [43, 44]. Thus, each cortical pulse was followed by a reinforcing light flash with a delay of 250 ms (Fig. **1B**). This value was chosen based on behavioural delayed reinforcement data [23]. At the outset, we were unsure which, if any, striatal neurons would be affected by this paradigm. We, therefore, thought it prudent to record a multi-unit response (Fig. **2**) to the cortical electrical stimulus within the motor territories of the striatum (Figs. **S1A**, **S2B**, **S3B**, and **S4B**).

### 3.1 Converging Afferent Signals are Required for Corticostriatal Potentiation

As predicted from previous work [43, 44, 52], the suppressive effects of urethane anaesthesia on visual sensory responding in the collicular deep layers also blocked all sensory reinforcement of cortically-evoked striatal activity (all visually-reinforced trials preceding time-0 in Fig. **2A**). However, following disinhibitory injections of bicuculline into the superior colliculus, local collicular neurons became visually responsive (Fig. **2C**: top), facilitating the relay of sensory signals to the striatum *via* the tecto-nigro-striatal and tecto-thalamo-striatal projections [43, 44]. Although collicular disinhibition enabled the light flashes to induce reliable visually-evoked local field potentials in the striatal territory receiving input from the motor cortex (Fig. **2C**: middle), flash-induced spiking in this part of the striatum was rarely observed (Fig. **2C**: bottom). In contrast, the visual reinforcer progressively enhanced multi-unit responses in the striatum evoked by continuing motor cortex single pulse stimulation (Fig. **2A** blue line and Fig. **2B**; repeated-measures ANOVA of group data, condition x time interaction, F12,84 = 3.6; *P* = 0.0002). This potentiation of corticostriatal transmission lasted for at least 1 h after the local disinhibitory effect of bicuculline had worn off – indicated by collicular neurons becoming unresponsive again to the visual stimulus. Representative examples of the facilitation of striatal multi-unit spiking activity caused by visual reinforcement are illustrated in Figs. (**2B** and **S5A-E**). Comparable potentiation of corticostriatal transmission was not observed when either the light flashes (Fig. **2A**: green line) or the disinhibitory injections of bicuculline (Fig. **2A**: red line) were omitted from the protocol. These control conditions confirmed first that visually-triggered reinforcing inputs to the striatum could not occur in the absence of signalling from the deep layer of the superior colliculus; and second, that the potentiation observed depends on the precisely timed visual stimulation as any non-specific activation caused by the general disinhibitory effects of intracollicular bicuculline were ineffective (cortical stimulation + collicular bicuculline – green line in Fig **2A**).

Further, to test the possibility that bicuculline-gated sensory reinforcement was having a general sensitizing effect in the striatum, unrelated to the electrically-evoked corticostriatal input, the electrical stimulation of the motor cortex was turned off during the period of sensory reinforcement. The cortical stimulation was reinstated when the SC stopped responding to the light flash. Potentiation of the striatal response was then significantly attenuated (Fig. **2D**: blue *vs*. yellow bars; Mann Whitney, U = 3, *p* < 0.02). Thus, a timed co-activation of cortical and sensory inputs was necessary to express sensory-reinforced potentiation of corticostriatal transmission fully.

However, due to the re-entrant looped architecture of the cortico-basal ganglia projections [53, 54], it is still difficult to ascertain the locus of plasticity *in vivo*. To exclude the possibility that sensory reinforcement was acting independently of transmission through the striatum, a further control experiment was conducted in which tissue surrounding the striatal recording electrode was temporarily inactivated by a local injection of the topical anaesthetic lidocaine during the period of sensory reinforcement. When cortically-evoked spiking in the striatum recovered from the local anaesthetic, the striatal response to cortical input was significantly depressed (Fig. **2D**: blue *vs*. purple bars; Mann Whitney, U = 0, *p* < 0.005). This attenuation was not due to a lack of recovery or to possible mechanical damage induced by the local injection of lidocaine as striatal spontaneous spiking after dissipation of the lidocaine effect was similar to that observed before injection (average baseline frequency count before lidocaine 31.7 ± 2.9 Hz *vs*. 29.1 ± 2.8 Hz after lidocaine; paired t-test, *p* > 0.1), while cortically-evoked response magnitude was reduced (before lidocaine 1.97 ± 0.13 *vs*. 1.28 ± 0.19 after lidocaine; paired t-test, *p* < 0.002). Subsequent analyses were conducted on data from each condition. The mean post-treatment magnitude of the striatal response (+44 to +56 min – the grey shaded area in Fig. **2A**) was compared with relevant data from the baseline period preceding treatment (-48 to 0 min). A reliable change from baseline was observed only when cortical stimuli were reinforced with light flashes presented during collicular disinhibition (Wilcoxon matched-pairs-signed-rank test Z = -2.366; *p* = 0.018). This increase in the amplitude of the cortically-evoked response was accompanied by a significant increase in its duration (Fig. **S6B**, Kruskal-Wallis, H = 26, d.f. = 4, *p* < 0.0001). although its latency was unchanged (Fig. **S6A**, Kruskal-Wallis, H = 4.5, d.f. = 4, *p* = 0.35). Together, the control experiments showed that the convergence within the striatum of cortical and sensory-evoked reinforcing inputs was necessary for corticostriatal potentiation to be observed.

### 3.2 Appropriate Signal Timing Required

A critical feature of behavioural reinforcement is that when a reinforcer precedes or is delayed too long after a causal action, its reinforcing effect is greatly diminished [23, 26, 38]. Consequently, to see if these principles also apply in the current model of corticostriatal plasticity, sensory reinforcement was presented at different times relative to the input to the striatum from the motor cortex. To accommodate an increased delay of the sensory reinforcement in this part of the study, the frequency of the cortical stimulation was reduced to 0.2 Hz (Fig. **1C**). Under these conditions and consistent with behavioural studies, significant potentiation was observed only when sensory reinforcement occurred within a limited temporal window (+250 and +1000 ms) following the signal from the motor cortex (Figs. **3** and **S5G** and **H**). Sensory stimuli presented before (-250 ms) or too long (2000 ms) after cortical stimulation were comparatively ineffective. Moreover, following the reduced number of reinforcement pairings presented during the period of collicular disinhibition in this protocol (recurrence of pairing 0.2 *vs*. 0.5 Hz), the magnitude of the potentiation effect was also significantly reduced (c.f. Figs. **2D** and **3**, for the +250 ms condition only; Mann Whitney, U = 10, *p* < 0.04).

### 3.3 Potentiation of Single-unit Activity

Next, we sought to explore ways in which the observed enhancement of the multi-unit response may be understood in terms of the effect of sensory reinforcement on the responses of single striatal units. When single pulse cortical stimulation (0.2 Hz) was coupled to sensory reinforcement (light flashes delivered +250 ms after the cortical stimulus) during collicular disinhibition, potentiation was observed in 8/11 recordings from single striatal neurons. From these data, the gradual increase in potentiation seen in the multi-unit response (Fig. **2A**) could be understood, in part, by the variable delays in the onset of the potentiation expressed by individual neurons (Fig. **4A**). Secondly, the potentiation of multi-unit spiking (Fig. **2**) was likely to reflect some neurons increasing their probability of firing at the same specific latencies at which they fired before potentiation (*e.g*., green neuron in Fig. **4**). Alternatively, other neurons would start responding to the cortical stimulation at new latencies, while at the same time maintaining similar spiking probabilities at pre-potentiation latencies (blue neuron in Fig. **4A**). Presumably, this variable pattern of firing latencies expressed by individual striatal neurons (Figs. **4B** & **C** and **S5F**) reflects a combination of distinct afferent corticostriatal and intrastriatal contacts. The short latency evoked striatal responses (< 12 ms) are most likely to be driven by monosynaptic cortical inputs [55], while the longer latency components (> 12 ms) probably reflect multisynaptic contacts. Interestingly, sensory reinforcement seems capable of modulating both mono- and multisynaptic inputs [35]. This, in part, would explain the overall pattern of potentiation we observed in our multi-unit recordings.

### 3.4 Multiple Sources of Plasticity

Appropriately timed phasic dopaminergic neurotrans-mission is considered an essential factor for the induction of corticostriatal plasticity [18, 21, 34]. To test this, we conducted our plasticity protocol in the presence of systemically administered D1-type (SCH23390) and D2-type (sulpiride) dopamine receptor antagonists. In preparation for interpreting the effects of dopamine antagonists before and after plasticity induction, we used vertically aligned 16-channel electrodes to record cortically evoked multi-unit activity within a larger area of striatal tissue (Figs. **S1B** and **S4B**). Because the channels extend 1.5 mm above the tip at a 10° angle, the recording sites of these 16 channel electrodes are more ventrolateral than suggested by the tip location and are likely sampling from a similar area to the other two experiments. After recording a pre-drug baseline control period (Fig. **S7C**), each subject was injected IP with either 1ml/kg of saline (0.9%; N = 4), the D1 dopamine receptor antagonist SCH23390 (0.2 mg/kg; N = 5), the D2 dopamine receptor antagonist sulpiride (30 mg/kg; N = 5), or an injection that contained both dopamine receptor blockers at the same respective concentrations (N = 5). A post-drug baseline period was then recorded, which included light reinforcement in the absence of collicular disinhibition (Fig. **S7C**).

To determine the effects of DA antagonists on baseline striatal responding [56] and to detect the subsequent presence of a potentiated response on single recording channels, we constructed post-stimulus time histograms for successive blocks of 120 cortical stimulations (Fig. **S7A**). When comparing the initial and drug baseline periods (blocks 1-4 *vs*. blocks 5-12 in Fig. **S7C**) we confirmed that the D1-type receptor antagonist reliably suppressed the striatal response to cortical stimulation (F = 28.55, *p* = 0.0001, using a randomisation test based on the F statistic [51]), while the striatal response was enhanced by the D2-type receptor blocker (F = 4.81, *p* = 0.0321 [51]; Fig. **S8**). When the DA antagonists were administered in combination, there was a small but reliable increase in baseline striatal responses (F = 9.71, *p* = 0.0015 [51]; Fig. **S8**). Finally, there were no reliable differences between the effects of dopamine antagonists on the baseline responses recorded on the electrode channels that would later potentiate, compared with those that did not (Fig. **S8**).

Since we were now sampling from multiple sites in the striatum, the next step was to determine for each animal how many of the multielectrode’s 16 channels could detect the cortically-evoked neural response. Typically, several adjacent channels were responsive, confirming the restricted striatal responsiveness patterns observed when moving a single electrode (c.f. Figs. **S4A** and **S4B**). Consistent with previous experiments, evoked responses comprised time-locked spiking increases that resolved into peak activity at fixed latencies (Figs. **S7A** and **S7B**).

**Fig. (6):**
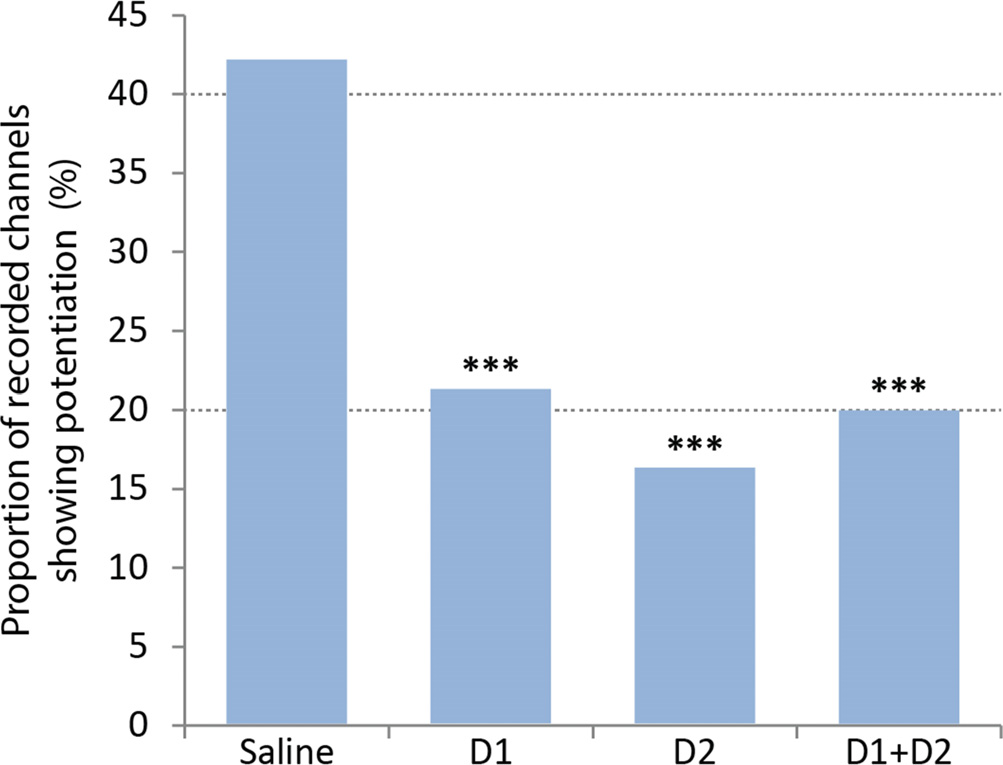
Effect of blocking dopamine neurotransmission on sensory-reinforced corticostriatal plasticity. Separate and combined blockade of D1-type and D2-type dopamine receptors reduced the proportion (%) of recorded channels showing potentiation (*** Chi-square = *p* < 0.001 compared with Saline group).

We then analysed the results from animals in which the two DA receptor blockers were administered separately by comparing the blocks' histograms following sensory reinforcement with the average histogram from a post-drug-baseline period (Fig. **S7B** and **S7C**). The main finding was that, compared with the saline control group, either DA receptor blocker significantly reduced the proportion of electrode channels on which potentiation was recorded (Fig. **6**; D1-type antagonist – Chi-Square 11.5, df = 1, *p* < 0.001; D2-type antagonist – Chi-Square = 15.9, df = 1, *p* < 0.001). However, on channels where it remained, the observed potentiation was largely unaffected by the DA antagonists; *i.e*., the mean duration, latency, and magnitude of the potentiation were not statistically different from the values obtained from the saline control group. Lastly, we determined the effects of a combined blockade of D1-type and D2-type dopamine receptors on the corticostriatal plasticity induced by sensory reinforcement. Compared with the saline control group, response potentiation was again observed on significantly fewer electrode channels (Chi-Square 11.6; df = 1; *p* = 0.001; Fig. **6**). However, the overall duration, magnitude, and latencies of positive instances of potentiation were again not reliably different from the saline control condition. We conclude that blocking D1-type and D2-type receptors effectively reduced but did not abolish the number of spatially distributed channels in the striatum on which sensory-reinforced potentiation could be observed.

## 4 DISCUSSION

The present study established an *in vivo* model of corticostriatal plasticity to explore the effects of delayed reinforcement signals generated by a natural sensory stimulus [13] and relayed into the striatum *via* intact afferent projections [43, 44, 46]. Validation of this protocol as an *in vivo* model of corticostriatal plasticity was strengthened after plasticity on behavioural time scales was observed. The study's main result was that a delayed light flash potentiated multi-unit striatal responses evoked by electrical stimulation of the motor cortex under experimental conditions known to promote visual sensory input to the striatum [43, 44]. The magnitude of the observed potentiation was quantitatively related to the number of stimulation-reinforcement pairings. Importantly, the observed potentiation of corticostriatal transmission was maximised when a behaviourally relevant time delay was imposed between input from the motor cortex and the sensory reinforcement. Reinforcement administered prior to or too long after the cortical input was ineffective. Therefore, this model of sensory-induced corticostriatal plasticity shares important aspects with the reinforcement that happens during behavioural conditioning [1, 23]. In both cases, an unexpected sensory event that occurs before an individual behavioural output cannot have been caused by the latter; therefore, the reinforcement process should not operate.

Similarly, an excessive delay between an action and a consequent reinforcing event invokes an increasingly difficult credit assignment problem, especially if irrelevant actions are expressed during the delay period. Thus, in our model and behavioural conditioning, effective reinforcement only occurs if a potentially contingent sensory reinforcer arrives hundreds of milliseconds after the neural representation of the causal motor output. This result, therefore, supports neoHebbian three-factor learning rules. It corroborates that motor-related input to the striatum generates a decaying synaptic eligibility trace that establishes a critical time window for reinforcement to induce potentiation [24-26, 38]. A mechanistic instantiation of this idea is provided by recent studies that have investigated the impact of delayed dopamine release on Hebbian plasticity at the corticostriatal synapse [33, 34, 36]. For example, in D1-type receptor-expressing medium spiny neurons, Yagishita *et al*. [33] showed that the structural plasticity of dendritic spines depended on the NMDA-receptor's sequential activation and dopamine D1-type receptor signalling pathways within a similarly restricted time window. Likewise, the potentiation of positive corticostriatal STDP by a delayed reinforcer in D1-type and D2-type receptors expressing striatal neurons was not observed if the activation of the dopamine inputs to the striatum [34] or the uncaging of dopamine [36] occurred with delays greater than ~2s after the corticostriatal pairing. The current protocol, therefore, offers a novel *in vivo* paradigm to evaluate the physiological, cellular, and molecular mechanisms underlying the concept of reinforcement eligibility [17].

The results show that the reinforcing effect of visual stimuli in the present study, under conditions where phasic DA is known to be released [44], occurred at subthreshold levels and in the absence of any changes in striatal spiking activity (Fig. **2C**). This could provide important insights into the mechanisms of sensory reinforcement during behavioural instrumental conditioning [57, 58]. However, to understand how this might be the case, it is necessary to appreciate that instrumental reinforcement operates to bias the selection of future actions (*i.e*., modulates the frequency with which reinforced actions are selected). Therefore, the mechanism(s) underlying behavioural reinforcement would be expected to be present within the neural systems responsible for action selection [5, 8, 57-59]. A recurring theme within basal ganglia research is that they constitute a mechanism within the vertebrate brain for selecting between competing behavioural motivations and actions [60-62]. The proposed selection mechanism is by selective disinhibition [63] within the parallel loop architecture of the basal ganglia [64, 65]. Instrumental reinforcement is thought to potentiate transmission in recently eligible (selected) channels, thereby increasing their probability of future re-selection [5, 57, 58, 66]. As ‘recently active channels’ cannot be predicted, reinforcement signals must be broadcast widely across the competing channels. It is, therefore, relevant that afferent projections likely to carry short-latency signals reporting the occurrence of an unpredicted sensory reinforcer (both nigro-striatal DA and thalamo-striatal GLU), project widely throughout the striatum [40, 67-69]. Within such an architecture, it is interesting to note in the current model of corticostriatal plasticity that sub-threshold reinforcer-driven depolarization [35, 43, 70] (Fig. **2C**), rather than an induction of all-out spiking, is preferred to adjust the sensitivity of recently active channels [66].

How sensory reinforcement might operate on the multiple cell types, and synaptic connections within the striatal microarchitecture will inevitably be complicated. The current *in vivo* model of cortico-striatal plasticity has revealed a complexity and diversity of potential synaptic changes. From our single-unit recordings of putative medium spiny neurons (MSNs), the observation that sensory reinforcement can potentiate existing responses (Fig. **4C** green trace) and induce spiking at previously unresponsive latencies (Fig **4C** blue trace) suggests the reinforcement process can operate at multiple synaptic locations and possibly across multi-synaptic pathways. This idea is reinforced by the finding that potentiated responses to cortical stimulation can occur at short latency (< 12 ms) but also at much longer latencies (up to 20-25 ms, Figs. **4C**, **S5**, and **S7C**). Potentiation observed in our multi-unit responses could result from changes in the intrinsic excitability of MSNs, dependent on D1-type receptors and A2a-receptor signalling [22, 34], but also from changes in synaptic transmission at glutamatergic synapses formed on MSNs [71, 72] and striatal interneurons [73, 74]. While the identification of the different striatal cell types was not the remit of the current test of whether any plasticity was detectable, a principle has been established where future studies using spike sorting from multichannel electrode arrays [75-77] can interrogate how sensory reinforcement can independently modulate components of intrinsic striatal microcircuitry.

Further, our results show that the point at which cortico-striatal potentiation can be observed following a period of sensory reinforcement is highly variable. Thus, some of the observed potentiations occurred soon after the reinforcement period had commenced, yet in other cases, it became evident only 40 to 60 min after its cessation (Fig. **4**). This is further evidence of a likely multi-dimensional response in mechanisms intrinsic to the striatum and possibly within other elements of the re-entrant looped architecture that connects the basal ganglia with the cerebral cortex. The current highly constrained model offers the opportunity to investigate independently how the different elements that contribute to the overall multiunit response are modulated by precisely timed sensory reinforcement [78].

Finally, our study confirms that plasticity induced in the striatum by delayed sensory reinforcement is partly dependent on intact DA transmission. Thus, some of the observed plasticity was blocked by systemic injection of a dopaminergic D1/D5-receptor antagonist [21, 22, 34, 79-81]. Some potentiations were also blocked by the systemic injection of a dopaminergic D2/D3-receptor antagonist. This latter effect could, in part, be attributed to the blockade of a form of long-term potentiation dependent on the activation of D2-type receptors and endocannabinoid-receptor signalling reported at the glutamatergic synapses formed on MSNs [71, 72]. However, in the condition where both D1-type and D2-type antagonists were administered, there was clear evidence that corticostriatal transmission could, in some cases, still be modulated by sensory reinforcement. The observed DA-independent plasticity might reflect spike-timing-dependent plasticity occurring at glutamatergic synapses formed on MSNs of the indirect pathway (t-LTP dependent on the activation of A2a adenosine receptors combined to the blockage of t-LTD dependent on D2R dopaminergic transmission) [9, 22, 34, 82]. To a lesser extent, the potentiation of cortical synapses formed on striatal GABAergic interneurons could be involved [73, 74]. In addition, thalamic gating by the light flash could have also contributed to the observed plasticity. The activated thalamic input may modulate the activity of fast-spiking interneurons [83] or cholinergic interneurons [41], with flow-on effects to corticostriatal inputs. In future studies, optogenetic methodo-logies will allow temporal control over the independent activation or silencing of the afferent pathways carrying sensory information to the striatum from the substantia nigra and/or the thalamus [59, 84-86]. Such studies will determine the relative importance of dopaminergic and glutamatergic transmission in sensory-reinforced plasticity within the striatal micro-circuit.

## CONCLUSION

The current *in vivo* model of sensory reinforcement offers a novel paradigm to address the physiological, cellular, and molecular bases of diverse forms of corticostriatal plasticity. An important feature of the paradigm is that it parallels significant aspects of instrumental conditioning in behaving animals [1, 17]. While caution must be exercised over the extent to which our results may have been influenced by the animals being anaesthetised, what we have been able to show is that when precisely controlled motor and sensory inputs converge on striatal units in a temporally relevant manner, the response to the motor input was potentiated. The extent to which this finding generalises to awake behaving preparations is a question for the future. That said, the fact that in our *in vivo* model, behaviourally relevant afferent projections [40, 42, 45-47, 59] can be appropriately activated by a natural sensory stimulus in a reduced anaesthetised preparation [43, 44] offers a degree of experimental control that would be more difficult to achieve in awake behaving animals. While the current study was always intended as a principal demonstration of sensory-reinforced striatal plasticity, having shown that it can occur with precise experimental control, numerous additional features of this novel paradigm could be investigated. For example, will the current sensory-reinforced plasticity operate in the associative and limbic territories of the striatum? To support the observed plasticity, what cellular and molecular processes occur in different striatal cell types? Can sensory reinforcement potentiate striatal activity generated in functional territories coding for sensory information [40]? Such territories could reinforce contextual information in which an action leading to an unexpected outcome occurs [10]. A different line of future research would test whether variables that influence plasticity in the current model have comparable effects on behavioural reinforcement learning, conversely, whether variables known to influence the acquisition of novel actions have similar effects in the present plasticity model. A better appreciation of the neural processes underlying reinforcement can only assist our interpretation of instances when it fails or becomes pathologically modified, as in aspects of Parkinson’s disease [87], schizophrenia [88], dystonia [89], and addictions [90]. Understanding may also be a prerequisite for discovering rational therapies for these debilitating conditions.

## Data Availability

The data that support the findings of this study are available from the corresponding author upon request.

## LIST OF ABBREVIATIONS

DA: Dopamine
GLU: Glutamate
MSN: Medium Spiny Neuron
RANOVA: Random effects ANOVA
STDP: Spike-timing-dependent Plasticity
