# Supplementary material for: Sensory Reinforced Corticostriatal Plasticity
Source: Curr Neuropharmacol. 2023 Aug 29;22(9):1513–27. doi: 10.2174/1570159X21666230801110359 (PMC11097983; doi:10.2174/1570159X21666230801110359)
Supplement: Supplementary file 1 [file CN-22-1513_SD1.pdf]

Supplementary Material

Sensory Reinforced Corticostriatal Plasticity

Nicolas Vautrelle[1,2,#], Véronique Coizet[2,3,#], Mariana Leriche[1,2], Lionel Dahan[2,4], Jan M. Schulz[1,5], Yan-Feng Zhang[1,6], Abdelhafid Zeghbib[2], Paul G. Overton[2], Enrico Bracci[2], Peter Redgrave[2] and John N.J. Reynolds[1,*]

[1]Department of Anatomy, Brain Health Research Centre, University of Otago, Dunedin 9054, New Zealand; [2]Department of Psychology, University of Sheffield, Sheffield, S10 2TP, UK; [3]Institut des Neurosciences de Grenoble, Université Joseph Fourier, Inserm, U1216, 38706 La Tronche Cedex, France; [4]Centre de Recherches sur la Cognition Animale, Université de Toulouse, UPS, 118 Route de Narbonne, F-31062 Toulouse Cedex 9, France; [5]Department of Biomedicine, University of Basel, CH - 4056 Basel, Switzerland; [6]Department of Clinical and Biomedical Sciences, University of Exeter Medical School, Hatherly Laboratories, Exeter EX4 4PS, United Kingdom

1875-6190/24 $65.00+.00 **© 2024 Bentham Science Publishers**

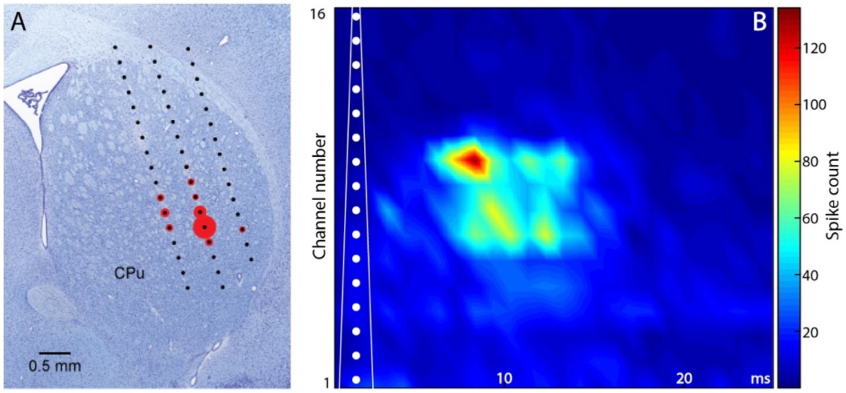

Fig. S1: Localized multi-unit responses evoked in the striatum by stimulation of motor cortex. **A.** An individual example of initial mapping used to establish the best location in the striatum for recording responses evoked by single pulse electrical stimulation of ipsilateral motor cortex. Responses were located in the lateral part of the striatum and extended approximately 1 mm in the rostro-caudal dimension. This information was used to guide probe placement in subsequent experiments. The size of red circles represents the relative magnitude of the evoked multi-unit response (see Fig 5). Abbreviation: CPu, caudate putamen. **B.** An individual example of peri-stimulus histogram obtained after processing striatal multi-unit responses to ipsilateral motor cortex stimulation recorded over 16 channels (120 stimulations/block).

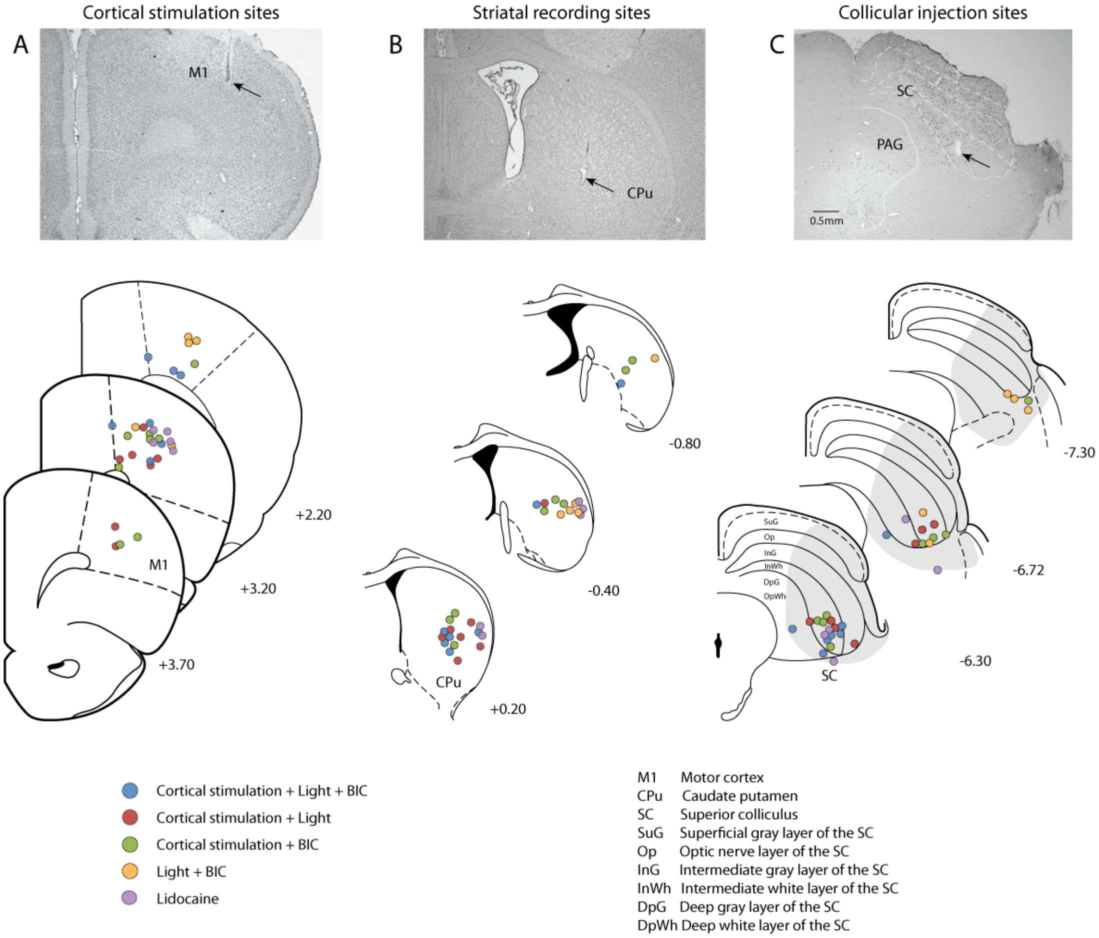

Fig S2: Reconstructions of stimulation, recording and injection sites from the first series of experiments. **A.** A photomicrograph of a typical stimulation site (arrow) in a section of motor cortex stained with cresyl-violet, and schematic representations of the stimulation sites for the different experimental conditions. **B.** A photomicrograph of a typical recording site (arrow) in a section of caudate putamen stained with cresyl-violet, and schematic representations of recording sites for the different experimental conditions. **C.** A photomicrograph of a typical injection site (arrow) in the superior colliculus and schematic representations of injection sites for the different experimental conditions. Note in the photomicrograph the distribution of neurons expressing Fos-like immunoreactivity in response to their activation by bicuculline and visual stimulation. A typical distribution of Fos-positive neurons following an injection of bicuculline into the superior colliculus is indicated by the grey shading in the schematic sections. The number associated with each section indicates mm relative to bregma.

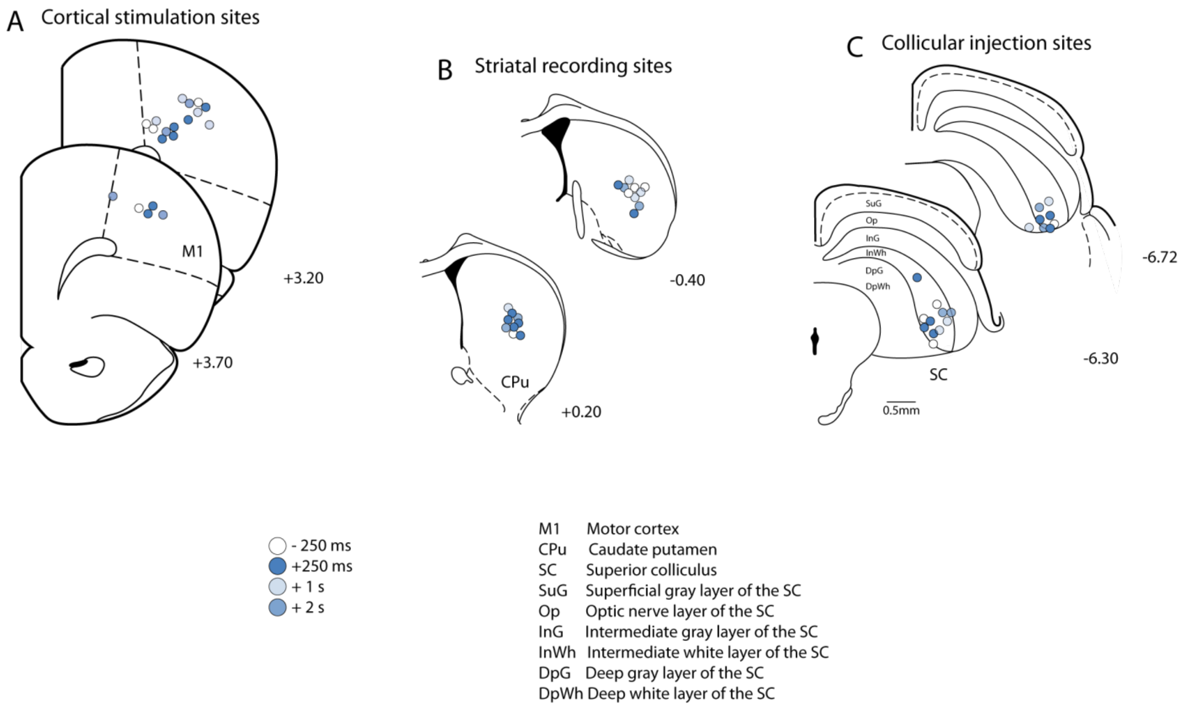

Fig S3: Reconstructions of stimulation, recording and injection sites from the second series of experiments. Schematic representations for the different experimental conditions of the stimulation sites in motor cortex **(A),** the striatal recording sites **(B)** and the collicular injection sites **(C).**

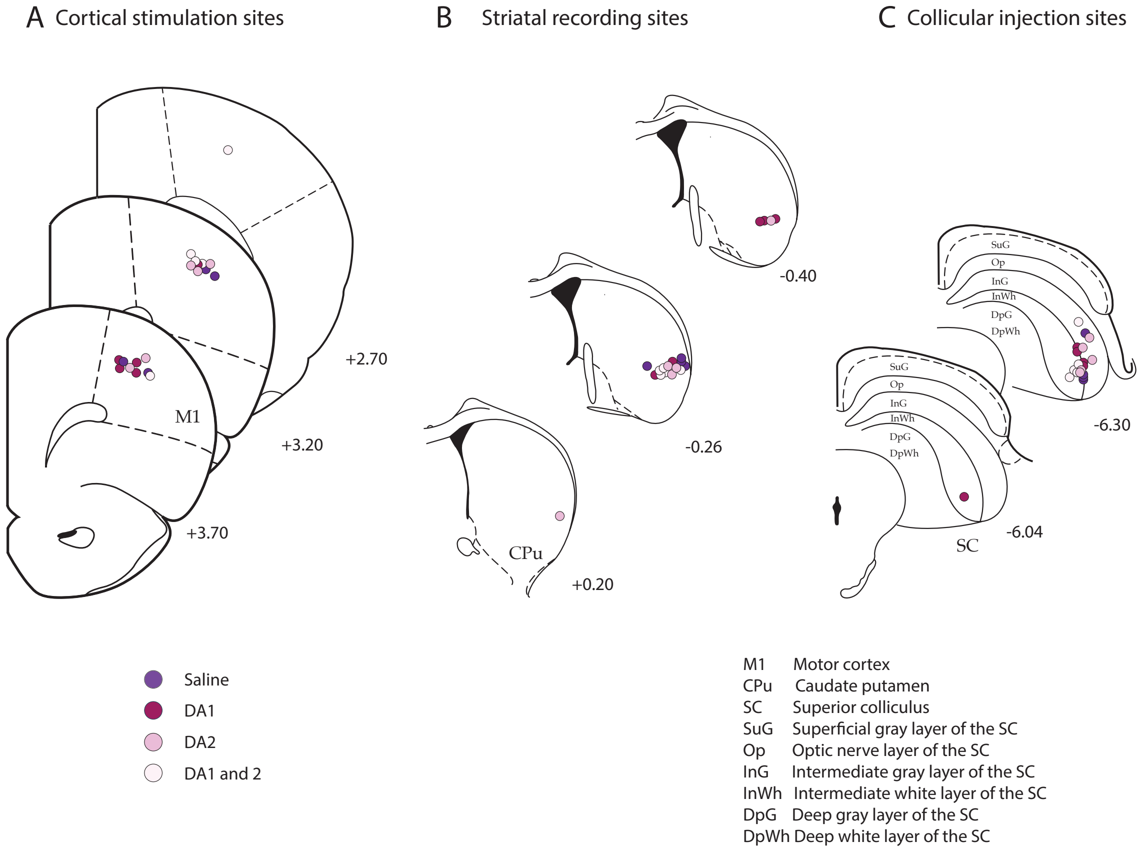

Fig S4: Reconstructions of stimulation, recording and injection sites from the third series of experiments. Schematic representations for the different experimental conditions of the stimulation sites in motor cortex **(A),** the striatal recording sites **(B)** and the collicular injection sites **(C).**

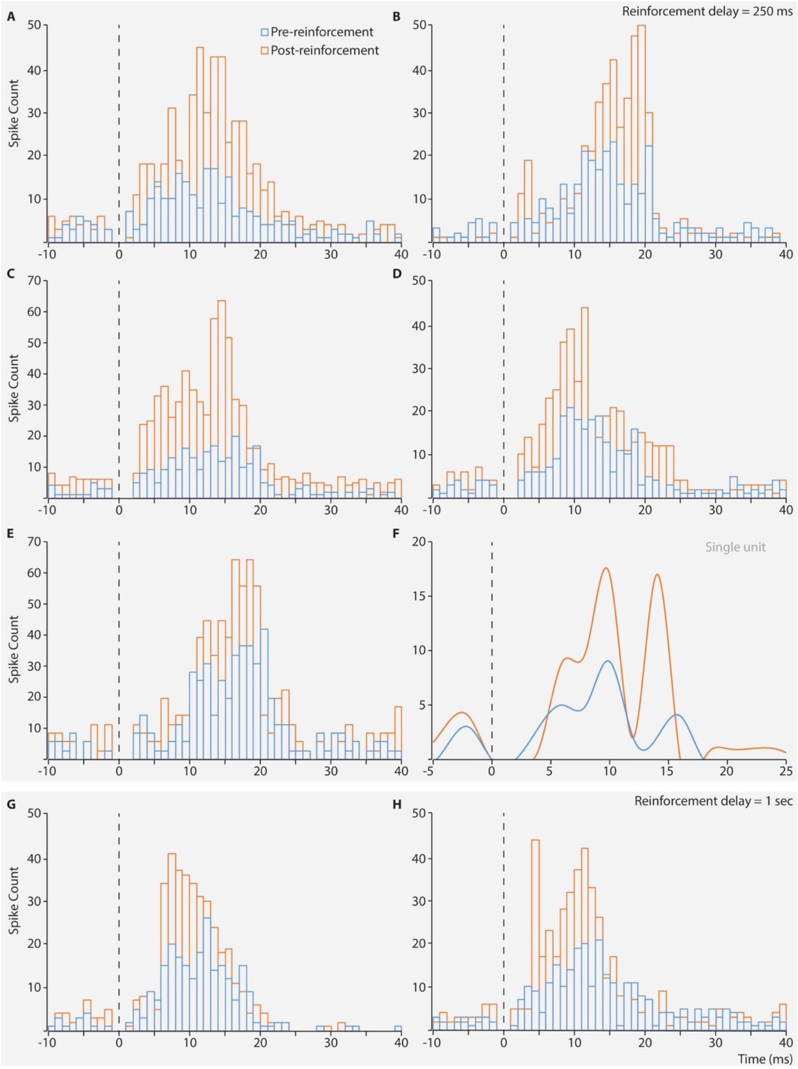

Fig. S5: Sensory-reinforced corticostriatal plasticity. **A-E.** Single case examples of striatal multi-unit potentiation induced by a delayed sensory reinforcer presented +250 ms after motor cortex stimulation. Comparison of peri-stimulus histograms obtained from pre-reinforcement and post-reinforcement recording blocks of 120 stimulations. **F.** Single case example of striatal single-unit potentiation induced by a delayed reinforcer presented +250 ms after motor cortex stimulation (60 stimulation blocks). **G-H.** Single case examples of striatal multi-unit potentiation induced by a delayed sensory reinforcer presented +1 sec after motor cortex stimulation (120 stimulation blocks).

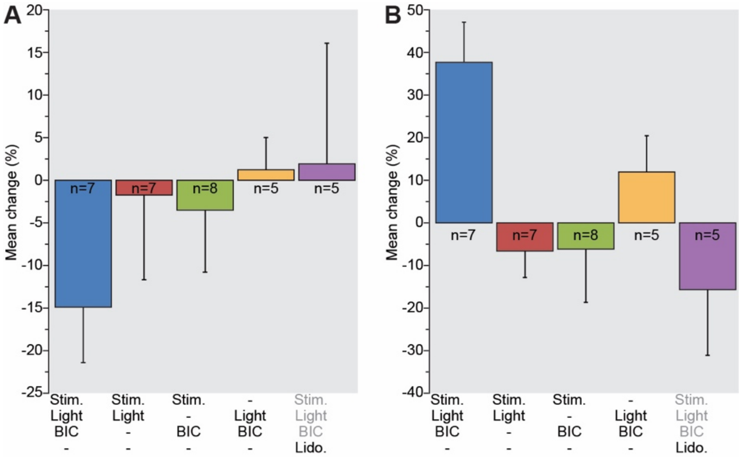

Fig. S6: Changes in corticostriatal response latency and duration. **A.** For all experimental conditions, mean change (%) in the latency of the cortical stimulus-evoked striatal responses 44-56min after collicular disinhibition (grey shaded area in Fig 2A). **B.** For all experimental conditions, mean change (%) in the duration of the cortical stimulus-evoked striatal responses 44-56min after collicular disinhibition. A significant increase in the duration of the striatal response accompanied the increase in response amplitude observed following collicular disinhibition.

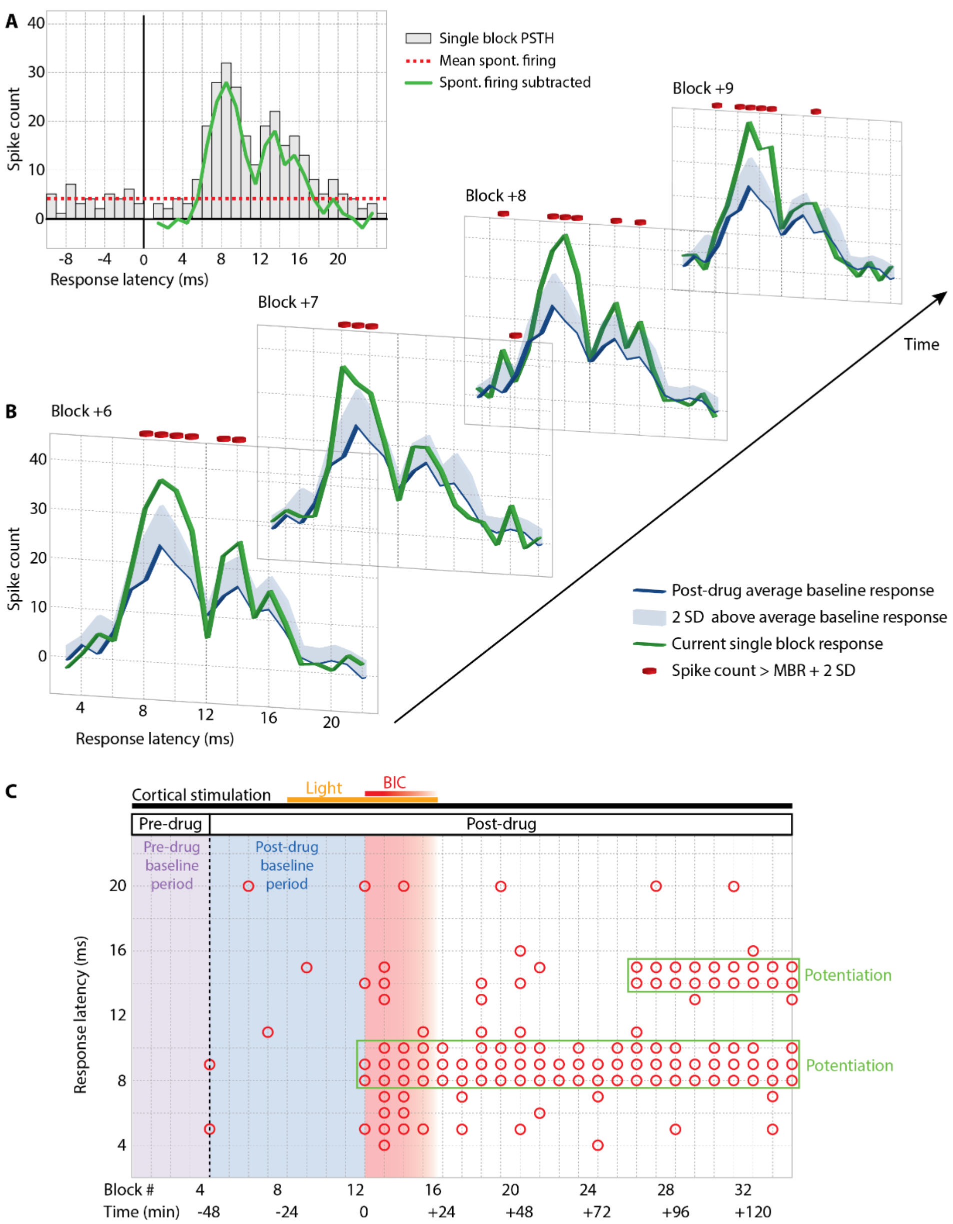

Fig. S7: Experimental protocol and data analysis of corticostriatal potentiation following the administration of dopamine antagonists. Striatal multi-unit responses to ipsilateral motor cortex stimulation were recorded simultaneously at different depths with a 16-channel electrode (NeiroNexus). This figure illustrates the classification of observed potentiation on a single channel following a period of sensory reinforcement (collicular disinhibition - BIC). **A.** Example of a single block (120 cortical stimulations) post-stimulus time histogram (grey bars) aligned to the cortical stimulus onset. For each PSTH, the absolute corticostriatal response (green line) was obtained by subtracting the mean pre-stimulus spontaneous firing (red dashed line) from the total response. **B.** Following the onset of sensory reinforcement (disinhibition of the superior colliculus with bicuculline) the histograms of successive block responses (green lines) were compared to the post-drug average baseline histogram (blue lines); this was calculated for each channel over the 8 blocks preceding collicular disinhibition. If the spike count value of any 1ms bin of the single block response was greater than the sum of the spike count value + 2 standard deviations (blue shading) for the same bin of the average baseline response (blue line), this bin was considered to be potentiated (red circle). **C.** Example of consistent potentiation (red circles surrounded by green boxes) observed within an experimental session. Single cortical pulses (0.33Hz) were presented throughout (top black bar). Following a pre-drug baseline period (4 blocks of 120 stimulations – purple shading) and halfway through the post-drug baseline period (blue shading), cortical stimulation was paired with a light flash (+250ms – yellow bar). This light flash was ineffective at inducing potentiation until the SC was disinhibited by local injection of bicuculline (BIC - red bar and shading). Peaks of potentiation (open red circles; compare with B.) were plotted against time with respect to the injection of bicuculine (x axis) and the response latency to cortical stimulation (y axis). An overall striatal response was classified as potentiated (green boxes) if potentiation peaks with similar latencies were detected: i) over a minimum of 5 consecutive blocks; or ii) over 7 or more consecutive blocks and such peaks were absent during the post-drug baseline period.

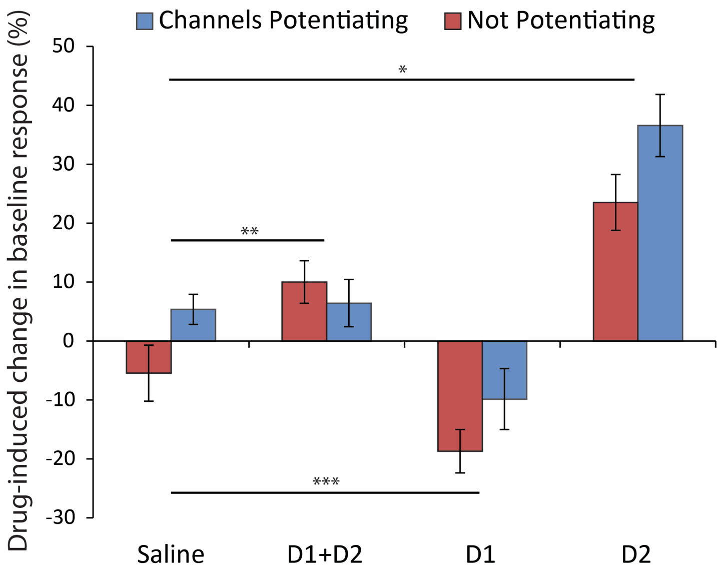

Fig. S8: Effect of DA antagonists on baseline striatal responses to cortical stimulation. Systemic administration of the D1-type receptor antagonist SCH23390 (0.2mg/kg) suppressed baseline striatal responding to the electrical stimulation of the motor cortex. Administration of the D2-type receptor antagonist sulpiride (30 mg/kg) enhanced the striatal response to cortical stimulation. When a combination of both antagonists was administered, a small but significant increase in the baseline striatal response was observed. In each condition, the effect of dopamine receptor antagonists on basal striatal responding did not differ between electrode channels that would later potentiate or not (randomisation test based on the F statistic[33], D1type receptor blocker: F=28.55, P = 0.0001; D2 receptor blocker: F=4.81, P = 0.0321; D1+D2 type receptor blockers: F=9.71, P = 0.0015).
